# A virtual culinary medicine intervention for ethnically diverse individuals with type 2 diabetes: development of the Nourishing the Community through Culinary Medicine

**DOI:** 10.3389/fnut.2024.1383621

**Published:** 2024-08-16

**Authors:** Lorena Macias-Navarro, John Wesley McWhorter, Diana C. Guevara, Sarah S. Bentley, Shreela V. Sharma, Jennifer H. Torres, David Ai, Natalia I. Heredia

**Affiliations:** ^1^Department of Health Promotion Behavioral Sciences, University of Texas Health Science Center at Houston, School of Public Health, Houston, TX, United States; ^2^Suvida Healthcare, Houston, TX, United States; ^3^Department of Epidemiology, Human Genetics & Environmental Sciences, University of Texas Health Science Center at Houston, School of Public Health, Houston, TX, United States; ^4^Baylor College of Medicine, Houston, TX, United States

**Keywords:** culinary medicine, type 2 diabetes, nutrition education, health promotion, cooking classes

## Abstract

Virtual culinary medicine education interventions have the potential to improve dietary behaviors, nutrition knowledge, cooking skills, and health outcomes for ethnically diverse individuals with type 2 diabetes. The purpose of this study is to describe the adaptation of the Nourishing the Community through Culinary Medicine (NCCM) program for virtual delivery, and the protocol for pilot testing this intervention. The intervention includes five 90-min virtual NCCM sessions streamed live from a Teaching Kitchen. Feasibility outcomes are recruitment, retention, acceptability, and satisfaction. Short-term effectiveness outcomes are measured through self-administered questionnaires, including perceived health, average daily servings of fruits and vegetables, frequency of healthy food consumption, shopping, cooking, and eating behaviors, cooking self-efficacy, diabetes self-management, perceived barriers to healthy eating, and nutrition knowledge. Demographics and biometric outcomes are sourced from the patient’s electronic medical records including glycosylated hemoglobin (HbA1c), Body Mass Index, and blood pressure. We will conduct a single-arm pilot study to test the feasibility and short-term effectiveness of NCCM program with individuals with type 2 diabetes.

## 1 Introduction

In the United States (U.S.), an estimated 37.3 million people (11.3% of the population) live with diabetes and approximately 90–95% of them have type 2 diabetes mellitus (T2DM) (1). T2DM is a chronic condition that arises either from unstable insulin production by the pancreas or the body’s ineffectiveness utilizing the insulin it produces (2). Insulin serves as a pivotal hormone responsible for regulating blood glucose levels (2, 3). The occurrence of hyperglycemia, also referred to as raised blood glucose, over the long term may lead to significant damage to various systems within the body, particularly the nerves and blood vessels (2–4). Diabetes-related complications, such as cardiovascular disease, kidney disease, neuropathy, blindness, and lower-extremity amputation, cause substantial morbidity and mortality among individuals with diabetes (5). Moreover, diabetes does not affect all sub-sections of the population equally; diabetes disproportionally burdens minority populations, with a higher prevalence in Non-Hispanic Black (12.1%) and Hispanic (11.8%) adults compared to non-Hispanic White adults (7.4%) (1).

To address this burden, many effective diabetes management strategies, such as intensive lifestyle modification programs, are widely implemented and accepted as being cost-effective (4). For example, there is substantial evidence that supports that consuming a healthy diet is a low-cost, effective treatment for managing T2DM (6–8). However, currently only 10% of the U.S. population adheres to the Dietary Guidelines for Americans (9–11). Moreover, there has been a decline in the diet quality of the U.S. population (12–14). Evidence suggests that diets that are high in ultra-processed foods and low in fruits, vegetables, and whole grains, coupled with low food literacy (i.e., inter-related knowledge, skills and behaviors required to plan, manage, select, prepare and eat food to meet needs and determine intake) (15) and a lack of cooking skills, may contribute to poor overall diet quality and the associated high national rates of T2DM (16–21). Furthermore, food insecurity, defined as the lack of access to nutritious and adequate foods, has also been associated with the intake of nutrient-poor foods and poor diet quality (22). It is estimated that 33.8 million people in the U.S. (roughly 10% of the population) are food insecure, with greater disparities among women, and Black and Hispanic households (23). Food insecurity has been related to lower self-efficacy in managing chronic conditions, such as T2DM, due to mental and financial stresses from increases in food, medications, and healthcare expenses (22, 24, 25).

To address the growing evidence of the detrimental effects of poor diet quality, low food literacy, limited cooking skills, and food insecurity for T2DM outcomes, culinary medicine interventions have recently emerged (15, 22, 24, 26–32). Culinary medicine is an evidence-based approach that combines the art and pleasure of cooking with nutrition science and medicine to increase healthy food consumption (33). Moreover, culinary medicine education interventions have been used to improve health outcomes in adults and offer the opportunity to manage and mitigate chronic conditions such as T2DM at the community level (10, 30, 34). This can be done by strengthening clinic-community linkages to offer lifestyle-based prevention services for patients, with the goal of teaching culinary skills, and providing hands-on experience preparing healthy foods in a collaborative group environment (30, 33).

Despite evidence suggesting culinary medicine interventions produce positive health outcomes, there are few combined cooking and nutrition education interventions designed for ethnically diverse individuals with T2DM (35). Given the disproportionate impacts of T2DM in various racial/ethnic groups, there is a need for inclusive and diverse nutrition interventions (36).

To bridge this gap, we developed the *Nourishing the Community through Culinary Medicine (NCCM)* program. The NCCM curriculum was adapted for synchronous virtual delivery from an in-person culinary medicine program, *A Prescription for Healthy Living (APHL)*, a collaborative effort between UTHealth Houston School of Public Health, Harris Health (the local safety-net healthcare system), and the Houston Food Bank (33). APHL offered a culinary medicine program to individuals with T2DM that were enrolled in the food prescription program, combining nutrition education with hands-on instruction to build cooking skills. The participant characteristics at baseline (*n* = 33 in the APHL group, *n* = 75 in the food prescription only group) were mostly female (80.0%) and Hispanic/Latino (86.2%) (33).

The framework for the NCCM curriculum draws upon constructs from *Social Cognitive Theory (SCT)* as a theoretical structure for understanding the factors that promote and maintain behavior change (37). The key SCT constructs used for NCCM include (1) outcome expectations of the taste of healthy foods, (2) knowledge of healthy eating, (3) self-efficacy for preparing healthy foods, (4) skills for preparing healthy foods, (5) perceived social support for consuming healthy foods, and (6) normative beliefs around eating healthy foods (38). Moreover, rather than only providing food prescriptions, this program provided electronic grocery cards to purchase cooking ingredients for class, coupled with live cooking instruction to create a collaborative learning environment for participants to engage and practice healthy eating behaviors through group experiential virtual cooking classes. Additional details regarding the SCT constructs used for NCCM can be found elsewhere (33).

The aim of this study is to (1) describe the development of the NCCM program for virtual delivery, and (2) detail the protocol for pilot testing the intervention with individuals with T2DM.

## 2 Materials and methods

### 2.1 Study population

In partnership with Sanitas Medical Center, a medical group practice and healthcare facility with locations in the Houston and Dallas regions, research staff recruited patients from Sanitas clinics that met the study inclusion criteria. The inclusion criteria were being an adult (18 to 70 years) with a T2DM diagnosis and HbA1c > 7.0, receiving care at Sanitas Medical Center clinics, and fluent in English or Spanish. Additionally, we screened for eligibility following these criteria: (1) having access to reliable internet and a device, like a cell phone, tablet or laptop, (2) someone in the household that was able to go grocery shopping before each class.

### 2.2 Recruitment

The study was conducted in accordance with the Declaration of Helsinki and approved by the Institutional Review Board of The UTHealth Houston (HSC-SPH-21-0555) on September 22nd, 2021. We started the rolling recruitment period from January 25th, 2022, to October 11th, 2022. Sanita’s care coordinators assessed individuals’ eligibility to participate and provided a printed flyer and sent a text message with a weblink to an online form for people who showed interest in the program. They completed the online interest form, sharing their contact information with UTHealth Houston. All recruitment materials were available in English and Spanish, and UTHealth Houston recruitment staff were fluent in both languages. After receiving their information, trained staff from UTHealth Houston contacted individuals via phone using an approved script for assessing eligibility and interest, and explained the program, including time commitment and potential advantages of participating in the program and minor risks while cooking at home (e.g., injuries with the use of knives, other cooking utensils, stoves, and ovens). After eligibility screening, participants provided written informed consent via REDcap (Research Electronic Data Capture), a secure, web-based application designed to support data capture for research studies (39). A bilingual staff member addressed any questions or concerns participants had regarding the program.

### 2.3 Development of the intervention

There are five main modifications that were made to adapt APHL for virtual delivery and NCCM implementation. First, the delivery format of the program was changed so that classes could be delivered through an online video conferencing platform (WebEx), with the main instructor hosting the virtual class live from the Nourish Teaching Kitchen at UTHealth Houston. One of the motivators for adapting APHL for virtual delivery was to reach a broader range of participants while COVID-19 restrictions (e.g., limitations on gathering in large groups) were still in place. Second, rather than providing food prescriptions, participants were provided electronic grocery cards via text message and email, to cover the cost of the ingredients and a shopping list for each class, which included tips on which items to choose to help participants with their shopping experience. These electronic grocery cards could be redeemed at any location of our chosen supermarket chain. Third, to further facilitate participant engagement and learning before and after class, we provided asynchronous virtual toolkit education content via text and email, which included links to access cooking skill videos, animated nutrition education videos, step by step recipes, shopping lists and kitchen set-up for each recipe, and nutrition and diabetes education handouts and worksheets. All of these materials were included in the study website; the development of this toolkit is described elsewhere (40). Participants used this toolkit to prepare for each culinary medicine session and as a reference post-session. Fourth, classes were offered during convenient times (usually after work hours, before standard dinner time) and the recipes were designed for a family of four. Fifth, cooking classes and toolkit learning materials were made more culturally inclusive (Table 1). To represent different ethnic groups, we specifically designed the intervention recipes to include a variety of cooking styles and flavors. For example, the stir fry was inspired by Asian cuisines, the turkey chili featured typical Hispanic/Latino flavors, and the pasta dish resembled more classic American cuisine. These recipes were pilot tested during the previously described APHL program (33). Additionally, the sessions were taught in two languages (English and Spanish).

**Table 1:** Topics and objectives for Nourishing the Community through Culinary Medicine sessions, a virtual culinary intervention among adults with type 2 diabetes.

Session	Learning objectives	Cooking technique	Topics and materials	Diabetes management focus	Social Cognitive Theory change objectives
1	Use safe and effective knife skills to cut a variety of vegetables. Discuss challenges with managing diabetes. Identify a healthy plate as ½ fruits and vegetables, ¼ lean protein, and ¼ whole grains. Understand how to set realistic goals. Learn how to prepare a balanced family meal.	Roasting vegetables and steaming grains.	Handouts: healthy eating; goal setting. Videos: my plate is your plate; small changes for health.	Introduce the balanced plate approach under the lens of creating blood sugar-friendly meals Introduce a sustainable behavior change aspect to diabetes management	Outcome expectations of the taste of healthy foods. Knowledge of healthy eating. Self-efficacy of preparing healthy foods. Perceived social support for healthy eating. Normative beliefs of healthy food.
2	Use safe and effective knife skills to cut a variety of vegetables. Describe what foods and beverages contain carbohydrates. Learn how to manage blood sugar levels using a MyPlate approach. Learn how to prepare a balanced family meal.	Cooking grains pilaf-style and baking.	Handouts: diabetes management; carbohydrate counting; label reading. Videos: all about carbohydrates; low blood glucose; high blood glucose.	Define carbohydrates Introduce symptoms and treatment of low blood sugar Introduce symptoms and treatment of high blood sugar	
3	Use safe and effective knife skills to cut a variety of vegetables. Practice strategies to develop healthy and mindful habits with family. Learn how to prepare a balanced family meal.	Soups, stews, and preparing dressings.	Handouts: healthy habits for the family Videos: a family affair why beverages matter.	Discuss how to eat a blood sugar friendly diet in the context of family meals Discuss the importance of selecting low/no sugar beverages for blood sugar management	
4	Use safe and effective knife skills to cut a variety of vegetables. Practice strategies for meal planning and grocery shopping. Practice reading food labels to encourage better food purchases. Prepare a balanced family meal.	Sautéing, stir frying, and steaming grains.	Handouts: planning healthy meals Videos: how to stock a healthy pantry; how to read nutrition facts labels.	Discuss grocery shopping and pantry staples to make blood sugar friendly eating more approachable Discuss how to read and what to look for in a nutrition facts label for carbohydrate management	
5	Use safe and effective knife skills to cut a variety of vegetables. Practice strategies to develop healthy and mindful habits to manage blood sugar. Describe plans to focus on health versus weight. Prepare a balanced family meal.	Sautéing, stir frying, and microwave cooking.	Handouts: mindful eating. Videos: beyond the scale mindful eating techniques.	Discuss mindful eating as an approach to counteract over-eating as an approach to improve blood sugar levels Discuss diabetes-related health markers to focus on	

### 2.4 Virtual culinary medicine education curriculum

This program consisted of five hands-on, 90-min virtual sessions. The sessions were facilitated by a native bilingual (English and Spanish) Registered Dietitian with culinary medicine training, with the aid of a co-teacher/assistant trainee. Kitchen setup was performed by the trainee, who additionally troubleshot technical issues (e.g., difficulties with accessing the video conferencing platform) and operated the kitchen studio cameras to alternate between a view of the instructor in the kitchen and an up-close, top view of the counter. This process allowed for lower staffing requirements for intervention delivery. The NCCM topics covered during the five sessions are presented in Table 1. Sessions were structured with a 15-min community connection and session introduction, 45–55 min of hands-on cooking, and 20–30 min of educational videos and facilitated group discussion. Participants were instructed on how to prepare their cooking station ahead of each class (i.e., have cutting board and chef knife ready, vegetables washed, and ingredients measured). Each session focused on two different cooking techniques widely shown to be the most important for improving healthy eating. Cooking videos explaining the different techniques were also provided ahead of time for participants to watch at their leisure and for reinforcement after sessions. During passive cooking time, 2–3 animated videos were played to facilitate discussion of the topics listed in Table 1. After each video, the instructor facilitated group discussion, with participants sharing their personal experiences and what they learned from or reactions to the videos. At the conclusion of each session, participants were encouraged to discuss likes, dislikes, recommendations for others, and how to personalize the recipes for their families – including adapting the recipe to fit cultural or individual preference. This conversation created additional ideas for creative ways to prepare food, driven by participants, to facilitate the incorporation of these recipes into their regular rotation. A printed toolkit with QR codes was mailed to participants’ homes that included all recipes and shopping lists along with handouts participants could fill out on their own time.

### 2.5 Study design

**Figure 1:**
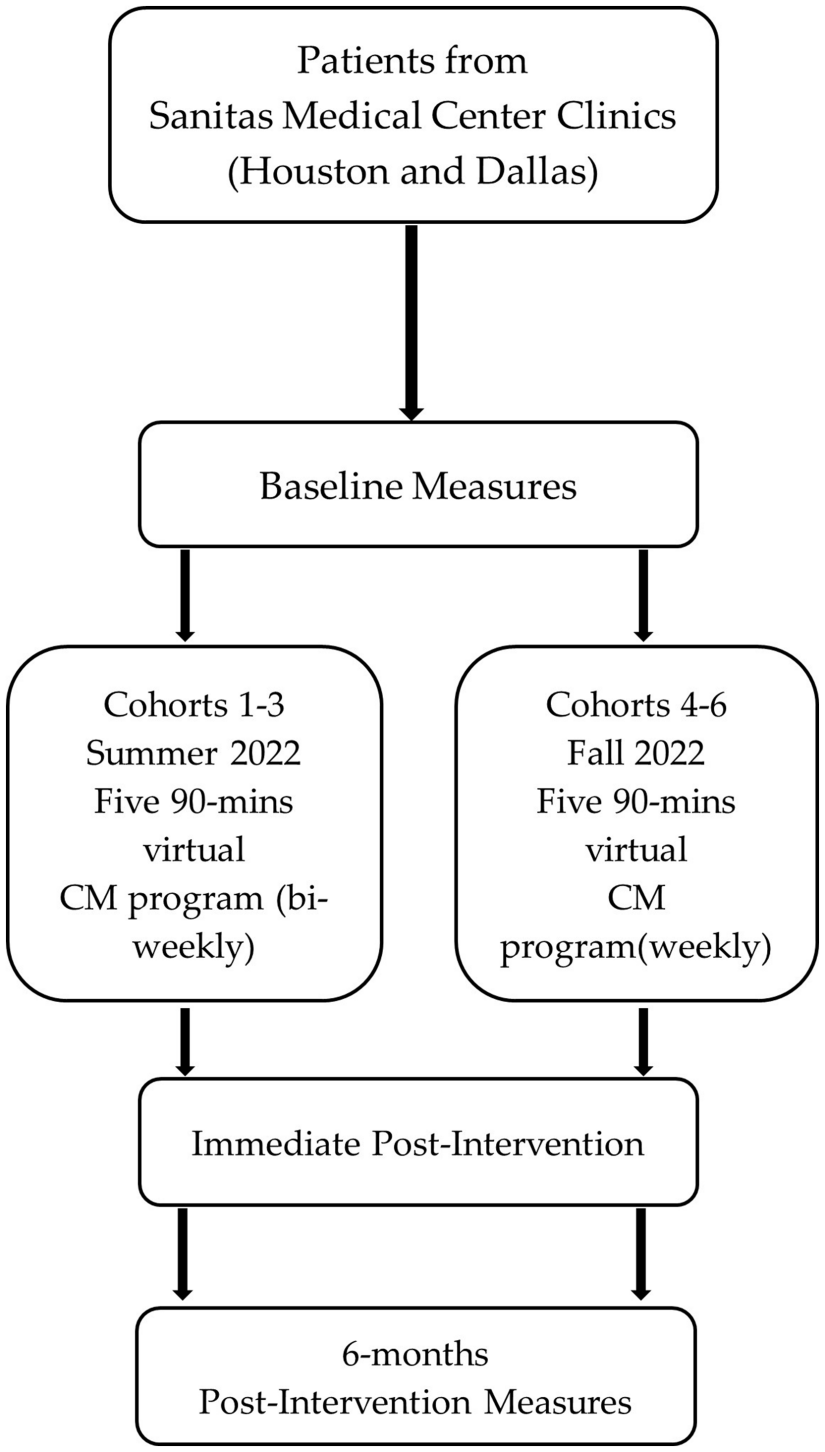
Nourishing the Community Through Culinary Medicine study design flowchart.

The study design was a single-arm prospective cohort intervention. The 6 cohorts (3 in English and 3 in Spanish) took place throughout 2022. The first three cohorts received the classes in the summer every other week. After implementing the program with these three cohorts, participants expressed an interest in having classes more frequently and we thought this might improve retention in the program. Thus, Cohorts 4–6, which were conducted during the fall, were conducted weekly, covering the same curriculum. We collected self-administered questionnaires at pre-test and post-test to evaluate participants’ dietary behaviors, nutrition knowledge, cooking skills and behaviors. Electronical medical record (EMR) data were collected at pre-test, post-test, and 6-month post-test (Figure 1). Additionally, we collected qualitative data from semi-structured interviews immediately post-intervention to evaluate patients’ experience during the intervention and satisfaction with the program overall.

### 2.6 Data collection

The partnership with Sanitas Medical Center sites allowed the use of participants’ EMR data to evaluate the impact of the NCCM program on biometric outcomes. Data were abstracted and shared by clinic coordinators with UTHealth Houston at three timepoints: pre-test (within 90 days before starting the NCCM program), at program completion (within 90 days of NCCM program completion), and 6-month post-test (within 90 days of 6-months completion of NCCM). These time measurements were chosen based on similar studies (41–45). Biometric outcomes included glycosylated hemoglobin (HbA1c), Body Mass Index (BMI), systolic blood pressure (SBP), and diastolic blood pressure (DBP). Additionally, demographics (age, sex, race/ethnicity) were sourced from the patient EMR records.

Participants were asked to complete a 15-min electronic questionnaire via REDcap at two timepoints: a pre-test questionnaire before the first class and a post-test immediately after the last session. Participants received a $25 gift card as compensation for their time for completion of each questionnaire ($50 total for study questionnaire completion). The pre-test and post-test questionnaires collected information on dietary intake, cooking skills, cooking-related psychosocial constructs, and diabetes management (details in the Measures section). Additional demographics were collected at pre-test including education, employment status, preferred language spoken at home, transportation, and participation in government assistance programs.

After each session, participants received a link to a virtual comment card in which they were asked to share feedback on the specific recipe, cooking method, and overall class flow. Participants voluntarily responded to the comment cards, and completion varied by participant. Each participant identified the class they attended (sessions 1–5) and then were asked to answer questions on how helpful they found the cooking techniques, class activities, and animated videos. Participants selected ‘yes’ or ‘no’ if they would make the specific recipe again on their own, if ‘no’ was selected then they were asked to explain in an open response. An additional comment section at the end encouraged participants to leave further feedback for improvement. These comments and feedback were not anonymous since the comment cards were linked to the participants’ IDs.

Qualitative interviews were conducted virtually by the research team at the end of the intervention to gather more robust information on the participants’ experience, identify areas for additional improvement in both the intervention and study design, and explain quantitative outcomes. Participants received a $20 gift card as compensation for their time after completing the interview. The semi-structured interview guide was adapted from the interview questions from a similar program (33) and was focused on assessing key feasibility and acceptability outcomes through open-ended, non-leading, and progressive questions (43–45). The topics included enrollment, engagement, experience with classes and resources, and comments regarding the program overall (Table 2). At the conclusion of the intervention, all enrolled participants were invited via text and email to take part in the virtual interviews. Depending on the preference of the participant, the interviews were conducted in either Spanish or English and all interviews were recorded. For English interviews, a transcription was performed by a smart note transcription service (Otter.ai) and inspected by staff for accuracy. For Spanish interviews, a transcription and translation into English was performed by bilingual research staff.

**Table 2:** Semi-structured virtual interview guide to assess feasibility and acceptability outcomes for the Nourishing the Community through Culinary Medicine culinary medicine program.

Topics	Main question
Enrollment	Let us talk about when you first learned about Nourishing the Community through Culinary Medicine: When did you complete the last class? How did you find out about the program? Probe: Did you see a flyer? Did a clinician recommend it? Tell me about why you were interested and enrolled in the NCCM program. Probe: How did you think NCCM would help you? Can you describe the sign-up process? Include steps like how the clinic told you about the program, forms you filled out, etc. Probe: Did you receive materials and instructions? What were they? Were they helpful? How? How was the program explained to you? I’m going to ask you a question on a 1 to 10 scale. On a scale of 1 to 10, with 1 being *not easy* and 10 being *very easy*, how would you rate the *ease of the NCCM sign up process*? Probe: Why did you choose that number? What suggestions do you have for how we can improve the sign-up process for the program?
Engagement	After the sign-up process was complete, think about the communication you received about Nourishing the Community through Culinary Medicine: What was your preferred form of communication (call, text, email) from our team? Probe: Were the messages frequent enough or too frequent? Were the reminder emails/texts helpful? Why? What suggestions or feedback do you have for how we can improve communication about the program?
Class & resource experience	Let us talk about the classes and resources: What suggestions do you have on how we can improve the virtual cooking classes? Probe: Were the classes too fast? Too slow? I’m going to ask you a question on a 1 to 10 scale. On a scale of 1 to 10, with 1 being *not easy* and 10 being *very easy*, how would you rate the ease of accessing the online meeting? Can you tell me about your experience visiting the toolkit website? I’m going to ask you a question on a 1 to 10 scale. On a scale of 1 to 10, with 1 being *not easy* and 10 being *very easy*, how would you rate the ease of the accessing the toolkits on the website? Probe: Was it easy to navigate? Could you find the recipes, shopping lists, and correct videos for each session? Which handouts did you find most helpful? What suggestions do you have on how we can improve the website or resources? What was your favorite recipe and why? What was your least favorite recipe and why? Where do you usually shop at to buy groceries? Probe: Was it more difficult for you to purchase groceries at Walmart? Why or why not? What are some challenges to healthy eating that you would like NCCM to address? Probe: In what ways was the program successful in addressing these challenges? In what ways was the program not successful in addressing these challenges? How did you initially feel about improving your cooking and eating habits? How do you feel about it now that you have participated in NCCM?
Program overall	And now, think about the overall NCCM program: How do you think NCCM has contributed to your cooking/eating habits? To your health? Probe: Can you give me an example of that? What is one thing that you’ll take away from the program? Probe: Can you tell me more about that? (if they give a one-word answer) What other suggestions do you have about the NCCM program? How can we better support you and others who want to eat healthier?

### 2.7 Measures

Feasibility outcomes were recruitment, retention, acceptability, and satisfaction (39). The feasibility of recruitment was determined by the number of individuals who were invited to participate by Sanitas staff, screened for eligibility, and then enrolled in the study. Records were maintained for all stages of recruitment in REDCap. Retention was measured by the proportion of participants who remained in the pilot study at the end of the intervention and completed post-test data collection. This was measured through attendance records. As recommended for pilot studies, *a priori* targets for recruitment and retention were set using relevant literature and the authors’ prior experience: > 60% of eligible individuals enroll, and ≥ 70% of participants are retained in the study (33). Acceptability and satisfaction were measured informally with comment cards to continuously engage with participants during the program and formally through in-depth interviews with participants post-test to understand participants’ experience and learning journey throughout the NCCM program.

Short-term effectiveness outcomes (Table 3) were measured through self-administered questionnaires. Perceived health was measured by 1 item validated survey with response options from *Excellent* (6) to *Very Poor* (1), with higher scores indicating better perceived health (46). Average daily servings of fruits and vegetables were assessed with 2 items (1 for fruits and 1 for vegetables), with response options from *None* (1) to *4 or More servings (6).* These items were adapted from a previously validated instrument to assess fruit and vegetable consumption (47). The two items were assessed separately, as well as summed, for a maximum score of 12, with higher numbers indicating a greater number of servings of fruits and vegetables (47). Frequency of healthy food consumption was measured by 7-item validated measure to assess typical food consumption behaviors, with response options from *Not at all* (1) to *More than once a day* (5); a sum was created, for a maximum score of 35, with higher numbers indicating greater frequency of healthy food consumption (48). Shopping, cooking, and eating behaviors were measured by 8 items, adapted from a previously validated survey, with response options from *Never* (1) to *Always* (5); items were summed for a maximum score of 40, with higher numbers indicating more ideal, healthy behaviors (48). Cooking self-efficacy was assessed by 10 items, not previously validated, at post-test only, five items that ask about self-efficacy before the program, and five ask about self-efficacy after the program, with response options of *Not sure at all* (1) to *Extremely sure* (5) (49). The scores were summed, and a higher score suggests greater self-efficacy for cooking. Diabetes self-management was measured by a 16-item validated scale, with response options from *Does not apply to me* (1) to *Applies to me very much* (4); a summed scale was created per standard protocol (50). Perceived barriers to healthy eating consisted of 9 items, not previously validated, with response options from *Strongly Disagree* (1) to *Strongly Agree* (5) for a maximum score of 45, with a lower number indicating fewer perceived barriers (48). And finally, nutrition knowledge was assessed with a single item, not previously validated, with response options providing different pictures of *MyPlate;* the correct answer was scored 1, and wrong answers were scored 0 (33).

**Table 3:** Short-term effectiveness outcomes of the Nourishing the Community through Culinary Medicine culinary medicine program.

Scales	# items	Example item	Response options	*Cronbach’s α
Perceived health	1	Overall, how would you rate your health in the past FOUR weeks?	1 (Very poor) - 6 (Excellent)	NA
Servings of fruits and vegetables	2	How many servings of FRUIT do you eat or drink each day?	1 (none) -6 (4 servings or more)	0.63
Frequency of healthy food consumption	7	How often do you typically eat a green salad?	1 (not at all) -5 (more than once a day)	0.66
Shopping, cooking and eating behaviors	8	How often do you plan meals ahead of time?	1 (never) - 5 (always)	0.74
Cooking self-efficacy	5	How sure are you that you can prepare fresh or frozen green vegetables (e.g., broccoli, spinach)	1 (not sure at all) - 5 (extremely sure)	0.86
Diabetes self-management	16	The food I choose to eat makes it easy to achieve optimal blood sugar levels.	1 (does not apply to me) - 4 (applies to me very much)	0.80
Perceived barriers to healthy eating	9	I do not eat fruits and vegetables as much as I like to because I do not know how to cook the vegetables	1 (strongly disagree) - 5 (strongly agree)	0.83
Nutrition knowledge	1	When thinking about preparing a plate of food, how much of your plate should be filled with fruits and vegetables?	3 picture options	NA

*Cronbach’s α values above 0.70 indicate acceptable internal consistency of the survey items. The higher the value, the stronger the reliability of the survey scale.

### 2.8 Analyses

Acceptability and satisfaction were assessed through analyses of in-depth interviews. Transcripts were uploaded in NVivo 12 (51), a qualitative data analysis computer software, and the analyses were conducted by two researchers independently. We used the framework approach (52, 53), involving three stages: data management, descriptive accounts, and explanatory accounts (53). During the data management stage, two research staff members familiarized themselves with the data, reading the interview transcripts, developing a coding index, and assigning codes for data analysis in NVivo 12. Twenty-seven percent of the transcripts were then double coded independently by another member of the research staff for quality control. Double coding resulted in 98.9% agreement. After transcript coding was complete, the researchers moved to the descriptive stage of analysis in which they identified associations between the codes and developed categories in which the data moved from anecdotal to more general themes. Finally, in the explanatory stage, researchers developed patterns within the themes, reflecting on the original data to ensure accurate representation of participant accounts.

We analyzed feasibility outcomes and demographic characteristics using descriptive statistics, including means and standard deviations or the observed number and percentage, as appropriate. Recruitment and retention outcomes were calculated as percent of eligible individuals enrolled and percent of participants completing questionnaires. We assessed changes from pre-test to post-test for questionnaire (perceived health, servings of fruits and vegetables, frequency of healthy food consumption, shopping, cooking, and eating behaviors, cooking self-efficacy, diabetes self-management, barriers to healthy eating and nutrition knowledge) and EMR data (HbA1c, BMI, SBP and DBP) using both unadjusted and adjusted multilevel mixed-effects models. We used linear, ordered, and logistic regression, as appropriate for each outcome. Models were adjusted for the individual as a random effect. Covariates were tested, and adjustments were performed when estimates changed by ≥10%.

## 3 Results

The pilot study has concluded. The results from analyses will be reported in a subsequent publication.

## 4 Discussion

Similar to other research protocol manuscripts (54, 55), this paper provides practical information about the design and presentation of a theory-based culinary medicine intervention, including the recruitment process, data collection, measures and recruitment and retention outcomes. For this study, we adapted a culinary medicine education intervention for virtual delivery to improve the accessibility of the program and potentially reduce potential barriers for attendance such as transportation time and costs (56). Our intervention has fewer sessions (5 total) than the average number of sessions of many other cooking interventions (8 sessions). However, this was intentional since we designed the intervention to be practical for replicating in real-world, clinical settings by making all intervention and instructional materials publicly available (57). Like several other cooking-related interventions, our curriculum was based on the Social-Cognitive Theory constructs (58). However, unlike many studies, our curriculum included recipes intended to appeal to a culturally diverse audience of mostly racial/ethnic-minority populations in the U.S. (58).

We also developed a culinary medicine virtual toolkit, which we anticipate can maintain participant engagement and learning beyond the program and reduce the burden of attending longer interventions. Virtual education can reduce culinary medicine instructor and staff burden by utilizing co-teachers/assistant trainees from other health care fields. This training could potentially broaden staff knowledge on nutrition education and skills related to culinary medicine, addressing the current lack of health care professionals (e.g., physicians) trained on these topics (24). We anticipate that virtual delivery also enhanced participants’ learning experience; by utilizing their own kitchen and equipment at home, we hope they were able to improve their specific skills and confidence for cooking *at home,* in the environment where they will need to maintain this behavior moving forward (28). Virtual delivery also eliminates the need for a larger teaching kitchen that can accommodate all participants. Rather, if there is a set-up for the instructor, with accompanying technological equipment, participants can use their own kitchens. This enhances the potential for NCCM to be more widely adopted by other health centers who may not have access to large teaching kitchens.

In NCCM, we provided participants with electronic grocery cards and a shopping list for each class via text and email, rather than following a food prescription model. While potentially adding additional tasks for participants, this design may have contributed to additional skill development, especially related to shopping for healthy items. Moreover, facilitated discussion led by the culinary medicine instructor encouraged participants to provide positive and negative feedback on their class experience, which offers the opportunity to personalize and center culinary medicine education on the participants.

Given the prevalence of adults living with T2DM in the U.S., culinary medicine education interventions that are tailored for ethnically diverse populations have the potential to build skills that will facilitate long-term T2DM management (30). Previous research on culinary medicine interventions has demonstrated significant improvements on behavioral and psychosocial factors such as dietary patterns, perceptions towards healthy eating, improved cooking skills, and self-efficacy for healthy cooking and eating (7–11). Additionally, culinary medicine education interventions offer the opportunity to mitigate chronic conditions such as T2DM at the community level by strengthening clinic-community linkages to offer nutrition-based services to participants in a collaborative learning environment (30, 33). To successfully implement an effective culinary medicine program, it is important to understand the barriers and facilitators for healthy eating among the population being served. Some barriers may include limited kitchen equipment and kitchen space, varying taste preferences and cravings, perceived lack of time, and the high cost associated with purchasing healthy food (30). Additionally, social inequities and intrapersonal factors such as culture, self-efficacy for preparing foods, food literacy, and social support are crucial to incorporate into culinary medicine interventions (7, 8, 11). While prior results for culinary medicine interventions offer promise in improving T2DM management outcomes, to our knowledge, our intervention is unique in being specifically tailored to be culturally inclusive for an ethnically diverse population.

There were limitations in the present study. These included that this study was a single-arm pilot study without a control group, limiting certainty that our findings can be solely attributed to our intervention rather than other events and trends simultaneously occurring in the community. Additionally, self-efficacy measures were not collected at baseline, and this may present some limitations of our results. The use of self-reported variables, including dietary intake, may have introduced reporting bias. Access to technology was also a limitation, particularly for participants with low technological literacy and/or lack of access to a device that connects to the internet. To address low technological literacy, we demonstrated how to access the virtual platform and online materials prior to the first session. With this initial training, most participants were able to overcome low technological literacy barriers. Lastly, we did not plan for collection of feasibility and acceptability data from the healthcare clinic and other staff involved with or delivering the intervention, which should be a consideration for planned data collection efforts in future trials.

Data collection and tracking participants’ data was conducted in collaboration with Sanitas Medical Center in the Houston and Dallas regions, which presented advantages and disadvantages. On one hand, some biometric information was missing as participants were not required to complete and the study did not pay for a follow-up visit at the clinic. Although data for analysis were obtained via our partnership with the health clinics, its completeness was limited. To improve completeness of the data in future work, additional funds would be needed to cover the costs for this bloodwork. On the other hand, having our partners collect biometric data through the EMR and securely transfer it to our research staff at UTHealth Houston was advantageous in terms of data collection and data security. This design is also more practical, as future dissemination and implementation of this program in clinical settings will likely involve the clinic themselves evaluating patient outcomes based on their available EMR data using standard metabolic health markers.

## 5 Conclusion

In this paper we describe adaptations to the Nourishing the Community through Culinary Medicine (NCCM) program for virtual delivery and the protocol for pilot testing the intervention. Subsequently, we will report on program implementation and the results of the pilot study testing feasibility, acceptability, and preliminary outcomes of the virtual synchronous NCCM program. We will use the findings and lessons learned from this pilot study to implement further culinary medicine education interventions as a strategy to address the needs of adults living with T2DM.

## Ethics statement

The studies involving humans were approved by the Institutional Review Board of The University of Texas Health Science Center at Houston (HSC-SPH-21-0555). The studies were conducted in accordance with the local legislation and institutional requirements. The participants provided their written informed consent to participate in this study.

## Author contributions

LM-N: Investigation, Project administration, Supervision, Writing – original draft. JM: Conceptualization, Funding acquisition, Investigation, Methodology, Project administration, Resources, Supervision, Writing – review & editing. DG: Investigation, Methodology, Writing – review & editing. SB: Investigation, Project administration, Supervision, Writing – review & editing. SS: Conceptualization, Funding acquisition, Methodology, Resources, Writing – review & editing. JT: Investigation, Project administration, Writing – review & editing. DA: Investigation, Project administration, Writing – review & editing. NH: Funding acquisition, Investigation, Methodology, Project administration, Resources, Supervision, Writing – original draft.
